# Circum-Mediterranean cultural heritage and medicinal plant uses in traditional animal healthcare: a field survey in eight selected areas within the RUBIA project

**DOI:** 10.1186/1746-4269-2-16

**Published:** 2006-03-24

**Authors:** Andrea Pieroni, Maria Elena Giusti, Caterina de Pasquale, Cinzia Lenzarini, Eleonora Censorii, María Reyes Gonzáles-Tejero, Cristina Patricia Sánchez-Rojas, Jose M Ramiro-Gutiérrez, Melpomeni Skoula, Chris Johnson, Anaya Sarpaki, Athena Della, Demetra Paraskeva-Hadijchambi, Andreas Hadjichambis, Mohammed Hmamouchi, Said El-Jorhi, Mohamed El-Demerdash, Mustafa El-Zayat, Omar Al-Shahaby, Zahia Houmani, Mekious Scherazed

**Affiliations:** 1 SCH Group, Department of Social Sciences, Wageningen University and Research Centres, Postbus 8060 6700 DA Wageningen, The Netherlands; 2 Medical Biosciences Research Focus Group, University of Bradford, Richmond Rd., Bradford BD71DP, UK; 3 Department of Art, Music and Performance, University of Florence, Via della Pergola, 48, 50121 Firenze, Italy; 4 Department of Botany, University of Granada, Campus Universitario de Cartuja, 18071 Granada, Spain; 5 Park for the Preservation of Flora and Fauna, Technical University of Crete, Plateia Ag. Markou, Terma Ag. Titou, 73100 Chania, Greece; 6 Agricultural Research Institute (ARI) of Cyprus, P.O. Box 22016, 1516 Nicosia, Cyprus; 7 Institut National des Plantes Médicinales et Aromatiques, B.P 6388 Rabat Institut, Rabat, Morocco; 8 Department of Botany, Mansoura University, 60 Al-Gamorhria St., 35516 Mansoura, Egypt; 9 Department of Agronomy, Agro-Veterinary and Biology, Blida University, Douirete Route de Soumaa, 09100 Blida, Algeria

## Abstract

During the years 2003–2005, a comparative ethnobotanical field survey was conducted on remedies used in traditional animal healthcare in eight Mediterranean areas. The study sites were selected within the EU-funded RUBIA project, and were as follows: the upper Kelmend Province of Albania; the Capannori area in Eastern Tuscany and the Bagnocavallo area of Romagna, Italy; Cercle de Ouezanne, Morocco; Sierra de Aracena y Picos de Aroche Natural Park in the province of Huelva, Spain; the St. Catherine area of the Sinai Peninsula, Egypt; Eastern and Western Crete, Greece; the Paphos and Larnaca areas of Cyprus; and the Mitidja area of Algeria.

One hundred and thirty-six veterinary preparations and 110 plant taxa were recorded in the survey, with Asteraceae and Lamiaceae being the most quoted botanical families. For certain plant species the survey uncovered veterinary phytotherapeutical indications that were very uncommon, and to our knowledge never recorded before. These include *Anabasis articulata *(Chenopodiaceae), *Cardopatium corymbosum *(Asteraceae), *Lilium martagon *(Liliaceae), *Dorycnium rectum *(Fabaceae), *Oenanthe pimpinelloides *(Apiaceae), *Origanum floribundum *(Lamiaceae), *Tuberaria lignosa *(Cistaceae), and *Dittrichia graveolens *(Asteraceae). These phytotherapeutical indications are briefly discussed in this report, taking into account modern phytopharmacology and phytochemistry.

The percentage of overall botanical veterinary taxa recorded in all the study areas was extremely low (8%), however when all taxa belonging to the same botanical genus are considered, this portion increases to 17%. Nevertheless, very few plant uses were found to be part of a presumed "Mediterranean" cultural heritage in veterinary practices, which raises critical questions about the concept of *Mediterraneanism *in ethnobotany and suggests that further discussion is required.

Nearly the half of the recorded veterinary plant uses for mammals uncovered in this survey have also been recorded in the same areas in *human *folk medicine, suggesting a strong link between human and veterinary medical practices, and perhaps also suggesting the adaptive origins of a few medical practices. Since most of the recorded data concern remedies for treating cattle, sheep, goats, and camels, it would be interesting to test a few of the recorded phytotherapeuticals in the future, to see if they are indeed able to improve animal healthcare in breeding environments, or to raise the quality of dairy and meat products in the absence of classical, industrial, veterinary pharmaceuticals.

## Background

On 17 October 2003, the *Convention for Safeguarding Intangible Cultural Heritage *was adopted in Paris at the 32nd Session of UNESCO [1]. In the Convention it was stated for the first time that *knowledge and practices concerning nature and the universe *are part of our cultural heritage. This means that ethnobotany, ethnobiology, ethnoecology (including ethnopedology and ethnoclimatology), traditional environmental knowledge, ethnoveterinary, folk medical, and pharmaceutical knowledge are now recognised as being inextricable components of culture, and therefore worthy of being protected and sustained (Pieroni et al., 2005a). The Convention's statement also signifies an important shift in the political approach to scientific research concerning *ethnobotany *and *traditional knowledge*, which in ethnopharmacology represent the focus or the starting point of much research and analysis.

In the Mediterranean region, there has been much theoretical dispute on the issue of commonality and difference in cultures, practices and social processes, especially within the entire debate on what, in cultural anthropology, is known as "Mediterraneanism" [2-8], i.e. the tendency of a few cultural anthropologists to automatically define a geographical area as an *homogenous cultural area *tout-court, and transform it into a stereotype.

In medical anthropology the issue of Mediterraneanism has often been addressed in the old debate on the "evil eye", however much less has been done to foster rigorous comparative perspectives in ethnobiology and ethnopharmacy.

In 2003 the EU Commission funded the research consortium RUBIA [9], within the subprogram INCOMED (International Cooperation with *non-European Mediterranean *countries). The main aims of INCOMED have been to improve research and cooperation on environmental issues, to better protect and conserve cultural heritage and the environment, and to improve health among all circum-Mediterranean countries.

The general aim of RUBIA has been to compare traditional plant usages in the circum-Mediterranean region, and among Mediterranean migrants in Central Europe.

The general scientific concept of this broad research consortium has been discussed elsewhere [10], but to recap very briefly, the specific objectives of RUBIA have been:

• To record ethnobotanical knowledge related to traditional plant uses in food, medicine, handicrafts, in the production of textiles, and for dyeing in eight selected areas of the Mediterranean;

• To develop an ethnographic knowledge database of all the recorded technologies and tools related to these plant uses;

• To deposit all these data in a centralised database and to compare them;

• To evaluate a few plant resources under the perspectives of their agronomic feasibility (cultivation of neglected or wild species in arid and semi-arid areas) and the small-scale eco-sustainable production of herbal products/phytotherapeuticals from local medicinal plants;

• To contribute to modern ethnobotanical and ethnographic museology by developing special sections in local botanical gardens and ethnographic museums that illustrate the recorded traditional uses of plants.

As part of this broad study, medicinal plant uses in *traditional veterinary practices *(ethnoveterinary) have been recorded in eight selected circum-Mediterranean areas. This limited set of data is the focus of this current paper.

Ethnoveterinary research has been defined as the "systematic investigation and application of veterinary folk knowledge, theory and practice" [11], and has recently been the focus of renewed interest in scientific debate and the formulation of animal healthcare policies in Europe and elsewhere, especially after recent dramatic emergencies such as bovine spongiform encephalopathy (BSE) in the UK, and the discovery of dioxin contamination in chicken meat in Belgium.

In many developing countries, plant uses in veterinary care have been the object of many field studies (see for example [12-22]; for an extensive bibliography, see [23]). Nevertheless, in Europe and particularly in the entire Mediterranean region, there have been relatively few specific studies on medicinal plant uses in ethnoveterinary practices [24-35].

Moreover, as yet no comparative fieldwork has been carried out on medicinal plants uses in traditional animal healthcare in the Mediterranean, despite the fact that interest in evidence-based veterinary phytotherapy is growing continuously in Western countries, due to an increased interest in complementary and alternative medicines, and also to the increasing numbers of veterinarians who are taking what is arguably a disputable common-sense view that phytotherapeuticals, with their fewer side effects, can be seen as suitable substitutes for a few allopathic pharmaceuticals in animal healthcare. Much more than pet healthcare is at stake here, because it is crucial that the management of sheep, cattle and goat breeding in the Mediterranean is improved dramatically in order to raise the quality of meat and diary products.

The aim of this paper, therefore, is as follows: to present the ethnoveterinary data that the research consortium RUBIA has gathered in the field in eight selected Mediterranean areas during the years 2003–2005, and to compare *veterinary plant uses*; to discuss the issue of medicinal plant uses under the perspective of a presumed common, circum-Mediterranean cultural heritage; and to briefly analyse the most interesting veterinary phytotherapeutical findings.

## Methodology

### Common ethnographic methodologies followed during the field study

The field methodological framework chosen for this research was that used in ethnobiology and ethnography [36-38]. The fieldwork was carried out in each of the chosen sites mainly by using participant observation and unstructured and semi-structured interviews that focused on details about traditional veterinary uses of plants. These include details on plant parts, the exact manipulation of the plants, their administration, their claimed use, and descriptions of the animals treated with specific folk taxa.

### Choice of the sites

During the consortium's first meeting, all the research teams involved in RUBIA adopted common criteria for selecting the local communities to be researched using field surveys.

Since very different countries were involved in the project, the main objective of the preliminary phase of the research project was first to define the criteria for selecting the study areas, and second to define the exact methodologies to be followed.

Since the main aim of RUBIA was to pinpoint and compare the different technologies, instruments and know-how related to mainly non-cultivated plants and their various traditional uses, it was decided that *rural *areas would be the best sites for carrying out the research. In this study, we have defined a rural area, not as a particular village, but rather as an expanse of land that shares similar geo-morphological features and ecosystems. The inhabitants chosen as informants did not necessarily have to belong to the same village, but could live in several nearby villages. Neither did they have to belong to the same ethnic group. Since one of the objectives of the field research was to investigate how traditional knowledge was passed through generations and from one area to another, it was important that the field research was carried out in a few diverse villages within the same region, as well as among diverse generations and gender groups.

**Figure 1:**
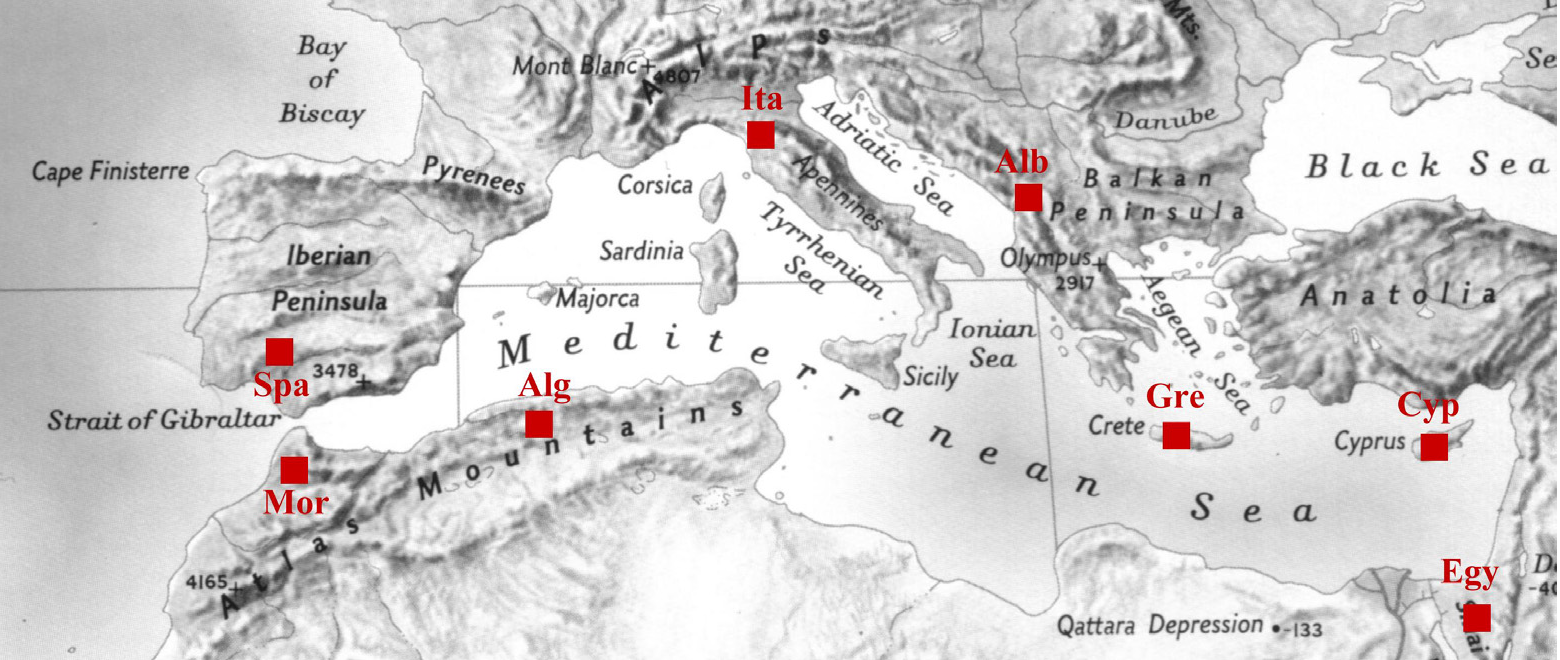
Geographical location of the selected study areas.

Field ethnobotanical studies were conducted from March 2003 through September 2005 in the following selected sites (Figure 1):

Albania: upper Kelmend Province;

Italy: the Capannori area (Eastern Tuscany) and the Villanova di Bagnocavallo area (Romagna);

Morocco: Cercle de Ouezanne, northern Morocco;

Spain: Sierra de Aracena y Picos de Aroche Natural Park (in the province of Huelva, south-western Spain);

Egypt: the St. Catherine area (Sinai Peninsula);

Greece: eastern and western Crete;

Cyprus: the Paphos and Larnaca areas;

Algeria: the Mitidja area, northern Algeria.

### Choice of the informants

Since the chosen methodology was purely ethnographic and not sociological in nature, the research teams at each of the sites used snowball techniques to select between 50 and 150 "knowledgeable" informants, without taking into consideration their gender or age ratios. Prior informed consent (PIC) was verbally obtained before commencing any of the interviews. Ethical guidelines adopted by the AAA/American Anthropological Association and by the ICE/International Society of Ethnobiology were rigorously followed.

All quoted plant taxa (with the exception of commonly cultivated plants) were gathered and identified by trained taxonomical botanists within each research area.

### Botanical references

Each field research team deposited specimens at the herbarium of the local university or research centre. Identification of the plant taxa was conducted following procedures outlined in standard taxonomic works:

• Flora Europaea [39];

• Flora d'Italia [40];

• Flora de Andalucía Occidental [41];

• Flora e Shqipërisë/Flore de l'Albanie [42];

• Flora of Cyprus [43];

• Flore de l'Afrique du Nord [44];

• Flora of Egypt [45];

• Nouvelle Flore de l'Algerie et des régions désertiques méridionales [46];

• Flore Practique du Maroc [47].

### Audiovisual documentation

Tape recordings were made during the interviews, and whenever possible video cameras were also used, and photographic documentation of all the recorded processes involving plants was highly recommended. This material has been used to produce a DVD and a RUBIA atlas, both of which are intended for a broader public audience and for political stakeholders rather than specifically for the scientific community.

### Database and data analysis

All the information gathered in the field has been managed using a centralized database hosted by the Greek institution. The structure of this database was conceptualised and designed by the RUBIA partners to include very specific information, however an exact description of the database design will not be provided here as it is outside the scope of this article.

Data analysis for this paper was carried out using very common software packages (Excel, SPSS) with the specific aim of comparing the parameters of plant taxa and veterinary plant usages.

## Results and discussion

### Ethnoveterinary practices in the selected sites

Knowledge of traditional health practices for animals is quickly disappearing in the study areas, as modern pharmaceuticals are replacing many plant remedies that have long been used to cure animals of various ailments. Particularly in the selected European sites including post-communist Albania, *institutionalised *capillary animal healthcare systems have been established in recent times in accordance with EU regulations. Even in the more remote areas pharmaceuticals are delivered to farmers and small-scale animal breeders, so the use of medicinal plants in these sites is likely to be linked to a nostalgic need to maintain familiar customs. A partial exception was found in the three selected areas in North Africa, where plant-based veterinary remedies are still being maintained. On the other hand, there is a new tendency among urban dwellers to use plant-based and homoeopathic remedies; however amongst rural dwellers it is generally only well-acculturated organic farmers and veterinarians who have moved back to the countryside from large cities who are involved in these practices. Nevertheless, indications of the drift towards plant-based and homoeopathic remedies were directly observed in the discussions with our informants in the selected areas (see the relatively low total number of plant taxa recorded, additional file: 1), even though "modern" veterinary phytotherapy was not part of the RUBIA focuses.

### Medicinal plants used in animal healthcare

In additional file: 1, we report on all the folk taxa recorded in the field survey that are used in local veterinary medicine. One hundred and ten plant taxa and 136 veterinary preparations have been recorded. In the same table, we give details of their administration, a description of their veterinary use and the animals that they are used on.

**Figure 2:**
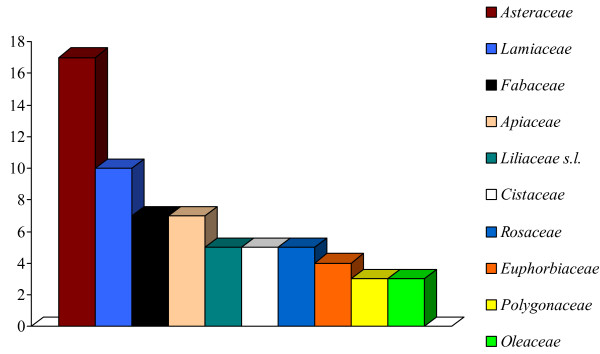
Most represented botanical families used in the folk veterinary phytotherapy of the selected sites.

In Figure 2, we depict the most-used botanical families overall, with Asteraceae and Lamiaceae heading the list. Another comparative work [48] has already pointed out that people across the northern hemisphere use certain taxa belonging to the same plant families for medicinal purposes. The Asteraceae family, for example, ranks in this analysis first in three of four of the selected regions and second in the fourth.

However, we believe that the predominant use of taxa belonging to a given botanical family in one area is very difficult to assess because of the large variety of plants that are available within one single region.

Following our analysis of the botanical family members used for veterinary purposes in each country, we found we could not speculate on Moerman's disputable proposition, since the areas we considered were very restricted and not at all *phytogeografically representative *of the entire flora of the relevant countries.

Moreover, exact data (and not mere estimates) on the existing flora within these restricted areas are in fact missing in the national taxonomical-botanical literature.

The relatively common use of Asteraceae and Lamiaceae within the worldwide medicinal plant panorama is not new, however, and could be due to phytochemical features. For example, the fact that Asteraceae contain mainly sesquiterpene lactones while Lamiaceae contain many essential oils implies that taxa belonging to these two families have generally a very marked taste (bitter in the case of Asteraceae and aromatic in the case of Lamiaceae). Other authors have suggested that this could have had a role in the selection of these medicinal plants by the first human groups [49-51].

### Newly recorded veterinary plants

On comparing our recorded taxa with the ethnobotanical literature available in PubMed, Web of Science, and all the aforementioned ethnoveterinary references, we found that a few of the taxa recorded in our survey have not previously been recorded (or it have been very rarely recorded) as veterinary plants. Moreover, they have rarely been investigated using modern phytopharmacology and phytotherapy.

**Figure 6:**
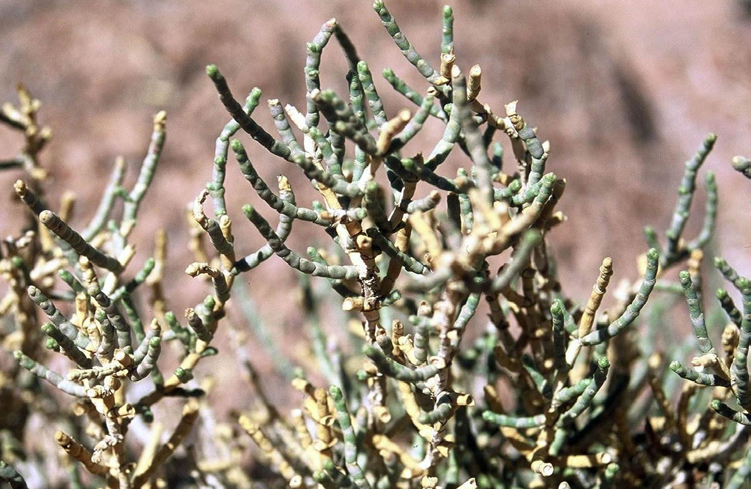
*Anabasis articulata *(Forssk.) Moq.

**Figure 7:**
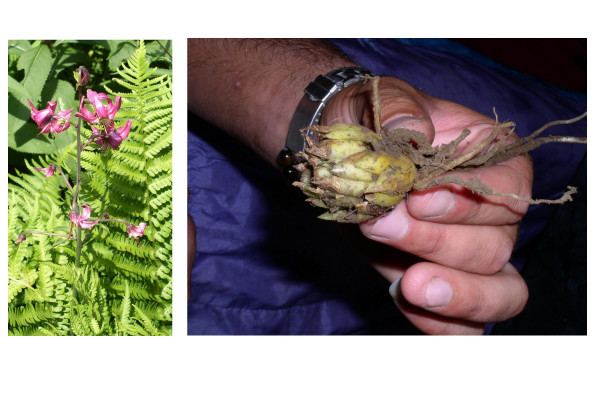
*Lilium martagon *L.

**Figure 8:**
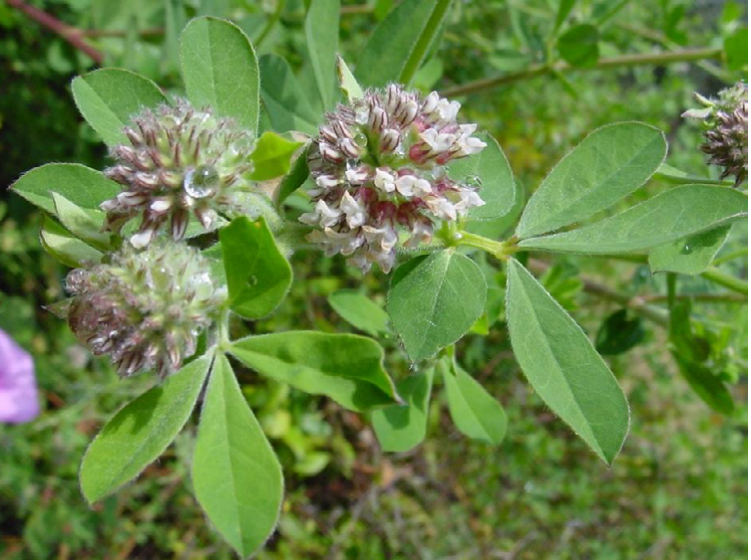
*Dorycnium rectum *(L.) Ser.

**Figure 9:**
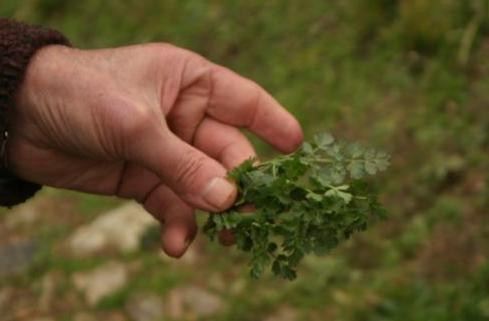
*Oenanthe pimpinelloides *L.

**Figure 10:**
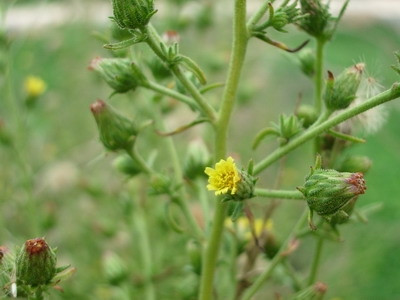
*Dittrichia graveolens *(L.) Greuter.

This was especially the case for the following species:

▪ the desert species *Anabasis articulata *(Chenopodiaceae, Fig. 6), which is used by the Bedouins of the St. Catherine area in the Sinai Peninsula in topical applications to treat animals with skin diseases. Larvicidal activity was found in an aqueous extract of this taxon [52];

▪ *Cardopatium corymbosum *(Asteraceae), whose roots are used in Cyprus in topical applications on wounds and as an antiseptic. Very few phytochemical works have been carried out on this species and its pharmacology is still largely unknown;

▪ *Lilium martagon *(Liliaceae, Fig. 7), whose bulbs are used to treat liver diseases in both humans and animals in Northern Albania [53]. In the ethnoveterinary literature, we found find that in northwestern Spain the same bulbs are used in the local ethnoveterinary practices, externally, as anti-inflammatory and analgesic [28];

▪ the legume, *Dorycnium rectum *(Fabaceae, Fig. 8), whose aerial parts were recorded in the Spanish site as being used as a decoction that is applied externally to treat burns and wounds. Apparently this species contains flavonoids, but its pharmacology has never been systematically investigated, except in a work by Molan et al. [54] on the activity of condensed tannins extracted from this species and used to treat nematoid motility;

▪ *Oenanthe pimpinelloides *(Apiaceae, Fig. 9), which is used as fodder in the Italian site, where it is thought to heal swollen stomachs in poultry;

▪ *Origanum floribundum *(Lamiaceae), recorded in the Algerian site where it is used to stimulate the appetite of cattle, sheep and horses;

▪ *Tuberaria lignosa *(Cistaceae), whose aerial parts are used in the Spanish site in a decoction to treat wounds in domestic animals; two works have very recently underlined the antiviral activity of this species [55,56];

▪ *Dittrichia graveolens *(Asteraceae, Fig. 10), whose aerial parts are used in Crete in an external application to treat lice in chicken. This species is well known for its essential oils, but its pharmacology is largely untapped.

### Origins of medicinal plant uses in veterinary treatments: the link with human medicine

**Figure 3:**
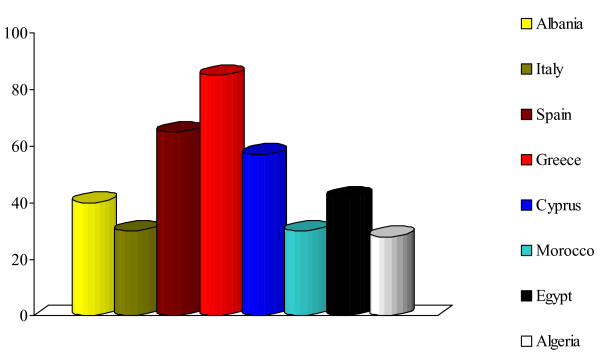
Proportion of the recorded veterinary plants used for healing mammals, which have also been recorded in the same selected study areas as being used in *human *folk medicine for treating "similar" diseases.

In the ethnoveterinary data recorded within the RUBIA project, nearly the half of the veterinary plant remedies for mammals has similar indications in local *human *folk medicine (Figure 3).

As discussed in other works [57,58], ethnoveterinary practices have probably followed two main evolutionary pathways: one based on the observations of self-medication in animals, and the other related to *human *folk medicine. Nevertheless, the relationship between human and veterinary practices has been complex and mutual. In some cases, humans could have tried certain plants on animals before applying them to themselves, but in other cases they undoubtedly used plants in veterinary practices, which were already used in traditional medicines to heal human beings.

Although it is difficult to distinguish between pharmacological and nutritional adaptations made by animals, there is certain evidence that animals deal with and take advantage of plant allelochemicals that have an apparent medicinal effect in a feeding context (see for example literature on self-medication in chimpanzees [59,60]).

### Comparative analysis: does Mediterraneanism really exist in medicinal plant uses?

**Figure 4:**
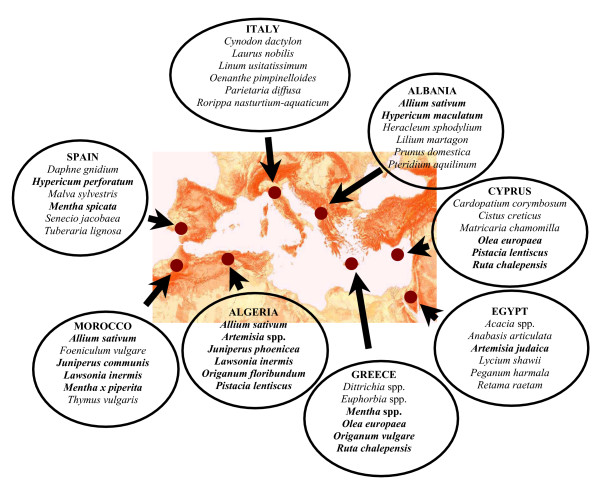
Most commonly recorded veterinary phytomedicines in the selected areas (in bold are reported taxa whose botanical genera have been recorded in at least one other country).

In Figure 4 we show the most quoted plant taxa used to cure animals in the various selected areas, and indicate in bold those taxa whose botanical genera have been recorded in veterinary practices in at least two areas. Overall, we found that there are ten botanical genera that are used in more than one country: *Allium, Artemisia, Juniperus, hypericum, Mentha, Lawsonia, Olea, Origanum, Pistacia,* and *Ruta*.

If we consider botanical genera that were quoted fewer times, we find that *Eurphorbia, Malva *and *Rhamnus *have also been recorded in more than one of the selected sites.

**Figure 5:**
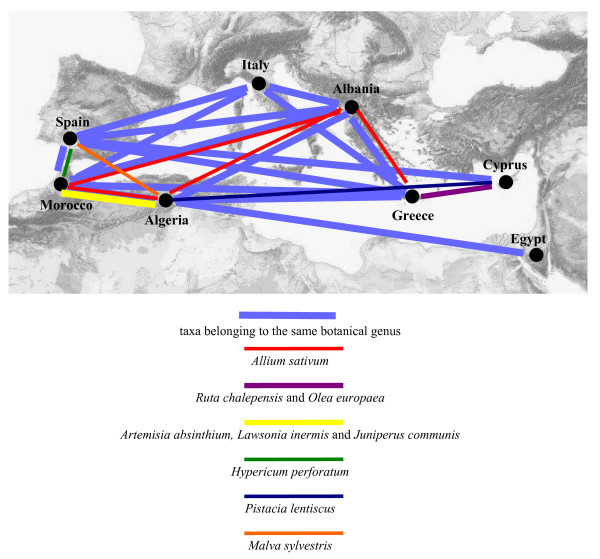
Diagram showing the botanical genera and taxa of veterinary folk remedies, whose use is shared in two or more of the selected areas.

Details about all the taxa and genera whose veterinary use is shared in diverse study areas are reported in Figure 5.

The percentage of taxa commonly used among the various areas has been extremely low (8%), however when all taxa belonging to the same botanical genera are considered, this portion increases to 17%. In the eastern sites (for example, Cyprus and in particular Egypt) there seems to be fewer taxa that are used in more than one area compared with the western sites. Hence the veterinary phytotherapy used in the eastern areas indicates that very few features are shared among the various regions (Figure 5). As far as the Egyptians are concerned, this could be due to the fact that the flora in the desert environment of the Sinai Peninsula is peculiar to that particular region.

In any case, looking at our overall results, we can see that any *common *heritage in plant uses in the Mediterranean would be extremely limited. It could be claimed that the chosen areas are not ecologically and culturally representative of the entire Mediterranean region or that much traditional ethnoveterinary knowledge has already disappeared, and this is truth. However it is may be worthwhile to emphasize that the sites were chosen by each research team individually, in accordance with common criteria. None of the other groups influenced their choice in any way, and so the data are basically bias-free.

On the other hand, it is clear from how the entire project has been conceived that our data are *not representati*ve either of the ethnobotany/ethnoveterinary of all the countries involved in this study. In our opinion, the data shown here should motivate instead other ethnobotanical research teams to carry out further comparative analyses in the Mediterranean. We also believe that our findings raise a crucial question concerning the assumption that *it has to exist *a common circum-Mediterranean cultural heritage that influences many human activities and the ethnobotany in the region. If it is true that during the entire history of this broad geographical area, a very complex system of exchanges and relationships between cultures has taken place (which must have had a remarkable influence on scholastic traditions in medicine and pharmacopoeias), we believe that the notion of a common cultural heritage in the Mediterranean *ethnobotany *and *folk knowledge *would represent, at least in part, a cultural construction. Our data demonstrate in fact that is very difficult to speak about a "Mediterranean ethnobotany" as a whole; instead we have a very variegate and composite Mediterranean made by many "Mediterraneans". Hence we feel that Herzfeld, the first scholar to complete a study of the "evil eye" in Greece [2], was correct in criticizing the idea of a unitary Mediterranean.

All in all, a common cultural heritage, which surely can be seen in other fields of human knowledge and practice, cannot be automatically recognized when looking at this specific portion of the ethnobotanical data that we have recorded.

### Veterinary phytotherapy in Mediterranean rural areas: which perspectives for the future?

An interesting dimension of the veterinary traditional knowledge that was recorded during our study concerns those plants cited for healing cattle, sheep, goats, and camels, and used to improve the quality of milk and dairy products.

Far more research is needed in this domain in order to provide an understanding of the effects of specific plants on animal health in general, and especially on the quality and quantity of meat and dairy products. An important potential long-term output of this study and other studies like it could be the development of eco-sustainable projects that have as a primary goal the use of plant-based remedies in traditional and new agricultural and animal breeding systems.

Such projects could also permit the controlled use of suitable phytotherapeuticals and extracts derived from plants, perhaps under the supervision of local veterinary services, which could add further value to local products in many "marginal" Mediterranean areas. However, to accomplish this, the strategic and political agenda of many national veterinary services might need to be changed slightly, since there seems to be a great deal of bias against *pluralistic *concepts of animal healthcare in the regions we have investigated.

## Conclusion

Many other in-depth ethnobotanical comparative studies will surely be necessary in the Mediterranean region, as well as in other part of the world, before we can gain crucial clues about commonalities and differences in medicinal plant usages among different cultures. In particular, our study has demonstrated that there is an urgent need for the documentation of TK related to the intangible cultural heritage concerning *traditional plant uses*, and that such a heritage is much more complex that we may think. Ethnoveterinary data in the Mediterranean region could offer an extraordinary background for conducting serious studies aimed at implementing clinical phytotherapy in animal healthcare and the use of plant-derived nutraceuticals, with the aim of improving the quality of animal-derived food products.

## Supplementary Material

Additional file 1 Table 1 Click here for file
