# Supplementary material for: Circum-Mediterranean cultural heritage and medicinal plant uses in traditional animal healthcare: a field survey in eight selected areas within the RUBIA project
Source: J Ethnobiol Ethnomed. 2006 Mar 24;2:16. doi: 10.1186/1746-4269-2-16 (PMC1447602; doi:10.1186/1746-4269-2-16)
Supplement: Additional file 1 — Table 1 [file 1746-4269-2-16-S1.pdf]

Table 1. Recorded veterinary uses of plants in the selected areas

Botanical taxon and voucher specimen code	Botanical family	English name	Country	Status	Part(s) used	Administration	Application	Animal(s) treated	Ethnoveterinary use(s)	Quotation frequency
Acacia nilotica L.<br>M 01	Fabaceae	Egyptian thorn	Egy	W	Fruits and leaflets	Decoction	I	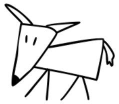	Digestive	xxx
Acacia tortilis Forssk.<br>M 02	Fabaceae	Umbrella thorn	Egy	W	Fruits, leaflets and gums	Decoction	I	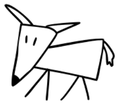	Digestive	xxx
Acer monspessulanum L.<br>LEP-ACE	Aceraceae	French maple	Alb	W	Wood	Burned, then the cold ashes are mixed with water and given the animals to drink	I	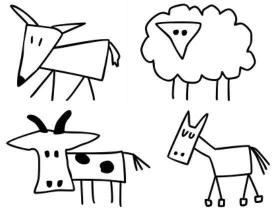	To treat the "evil-eye"	x
Achillea fragrantissima (Forssk) Sch. Bip.<br>M 03	Asteraceae	Lavender cotton	Egy	W	Whole plant	Decoction	I	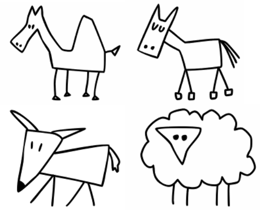	Digestive	xx
			Egy			Topic application	E	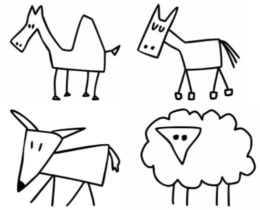	To treat skin diseases	xx

Allium cepa L.	Liliaceae s.l.	Onion	Mor	C	Bulbs	Fodder (seven onions)	I	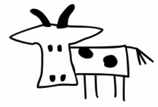	To facilitate the expulsion of the placenta	xxx
			Gre	C	Bulb	Suppository	I	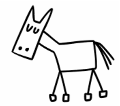	Digestive	x
			Gre		Bulb	Fodder	I	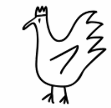	To treat lice and "skin bugs"	x
Allium sativum L.	Liliaceae s.l.	Garlic	Mor	C	Bulb	Fodder (mixed with human urine)	I	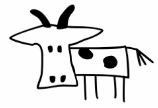	Digestive	xx
			Alg	C	Bulb	Fodder (mixed with oil oil)	I	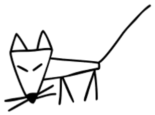	Vermifuge	xx
			Gre	C	Bulb	Plaster	E	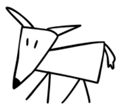 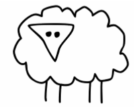	To treat skin diseases and wounds	x
			Alb	C	Bulb	One garlic clove is wrapped in a very small piece of cloth, together with two coffee beans and two grains of salt	E	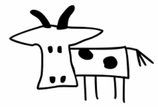 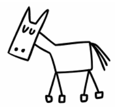	Hung on the horn of the animal in order to prevent the evil eye (*syni i keq*)	xxx
Althaea officinalis L.	Malvaceae	Mallow	Mor	W	Whole plant	Wrapped in linen and placed in the oral cavity of the horse	E	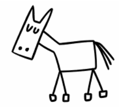	To treat oral inflammations	xx

Anabasis articulata (Forssk.) Moq.<br>M 39	Chenopodiaceae	Berry-bearing glasswort	Egy	W	Aerial parts	Topic application	E	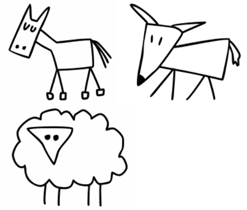	To treat skin diseases	xxx
Angelica archangelica L.	Apiaceae	Angelica	Mor	C		Macerate in vinegar (together with garlic)	I	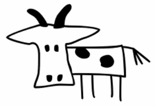	Digestive	x
Artemisia absinthium L.	Asteraceae	Wormwood	Mor	C	Leaves	Decoction	I	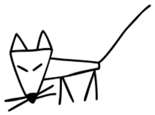	Anti-parasitic, Digestive	x
			Alg	W	Aerial part	Fodder	I	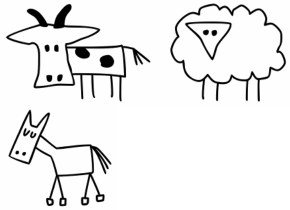	Digestive; to heal nervous diseases	xxx
Artemisia herba-alba Asso<br>AsAhe01	Asteraceae	White artemisia	Alg	W	Aerial parts	Fodder	I	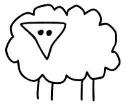	Vermifuge	xxx
			Alg			Decoction	I	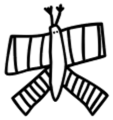	To treat "bees diarrhoea"	xxx
			Alg			Smoke from the burning plant	I	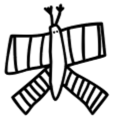	Acaricide	xxx
Artemisia judaica L.<br>M 07	Asteraceae	Judean wormwood	Egy	W	Aerial parts	Decoction	I	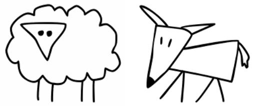	To heal urinary disorders	xxx

								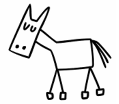		
Asparagus acutifolius L. LiAca01	Liliaceae s.l.	Wild asparagus	Alg	W	Leaves	Decoction	I	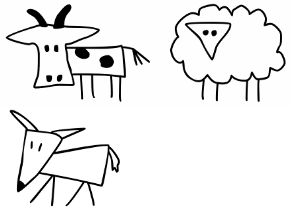	To treat stomach disorders	xxx
Astragalus lusitanicus Lam. GDA 49187	Fabaceae	Iberian milk-vetch	Spa	W	Aerial parts	Decoction	E	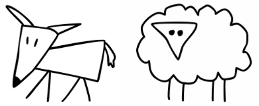	To treat skin diseases	x
Atraphaxis spinosa L. M 09	Polygonaceae		Egy	W	Aerial parts	Decoction	I	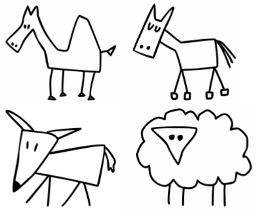	Digestive	x
Atropa bella-donna L.	Solanaceae	Deadly nightshade	Mor	C	Leaves, root and flowering head	Decoction	I	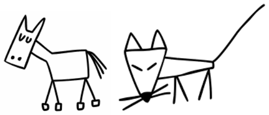	To stop coughing and reduce bronchial secretions	
Bufonia multiceps Decne. M 41	Caryophyllaceae		Egy	W	Entire plant	Fodder	I	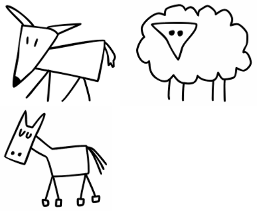	Digestive	x
Buxus sempervirens L.	Buxaceae	Box	Mor	W	Leaves	Crashed in external applications	E	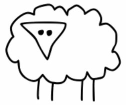	To treat skin inflammations	x

Calotropis procera Ait.<br>M 12	Asclepiadaceae	Giant milkweed	Egy	W	Whole plant	Decoction	I	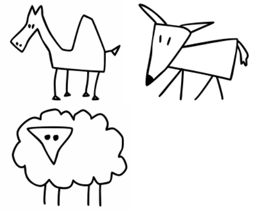	Digestive	xx
			Egy			Topic application	E	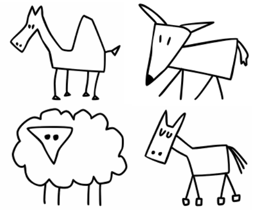	To treat skin diseases	xx
Cardopatium corymbosum (L.) Pers.	Asteraceae	Black chamoeleon	Cyp	W	Roots	Crushed and applied topically	E	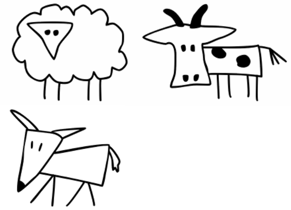	To treat wounds, antiseptic	xx
Carum carvi L.	Apiaceae	Caraway	Mor	C	Fruits	Crushed in topic application	E	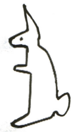	To treat skin lesions	xx
Centaurea alba L. ssp. *tartesiana* Talavera<br>GDA 49212	Asteraceae		Spa	W	Leaves	Decoction	E	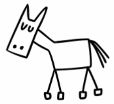	To treat wounds and callus (hyperkeratosis)	x
Cistus creticus L. and<br>*C. monspeliensis* L.	Cistaceae	Rock rose	Cyp	W	Exudate	Poultice	E	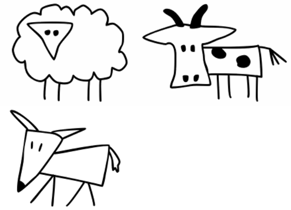	To treat skin disorder and belly aches	xx

Cistus ladanifer L.<br>GDA 49097	Cistaceae	Labdanum	Spa	W	Young stems	Topic application	E	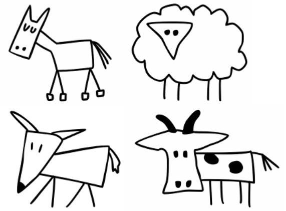	To heal bruises	xx
Cistus populifolius L.	Cistaceae		Spa	W	Leaves	Decoction	E	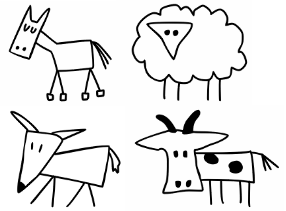	To heal wounds	xx
Cleome droserifolia (Forssk.) Delile<br>M 16	Capparaceae		Egy	W	Aerial parts	Decoction prepared from the dried aerial parts	I	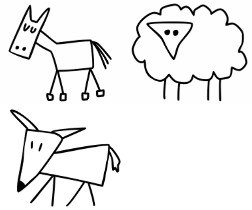	Digestive	x
Colchicum autumnale L.	Liliaceae s.l.	Autumn crocus	Mor		Fruits	Decoction	I	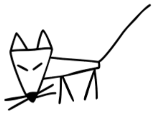	To treat rheumatoid arthritis	x
			Mor					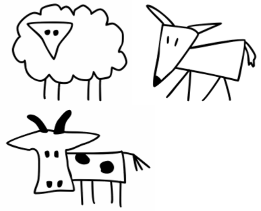	Carminative and digestive	x
Cucurbita pepo L.	Cucurbitaceae	Pumpkin	Mor	C	Seeds	Fodder, mixed with honey and castor oil	I	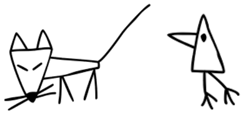	Digestive	x
Cynara scolymus L.	Asteraceae	Artichoke	Mor	C	Roots	Decoction	I	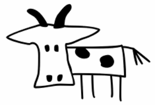	Digestive	xx

Cynodon dactylon (L.) Pers.	Poaceae	Bermuda grass	Ita	W	Entire plant	Fodder	I	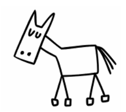	To treat intestinal inflammations	xxx
Daphne gnidium L. GDA 49213	Thymelaeaceae	Flax-leaved daphne	Spa	W	Bark	Braided and tied on the neck	E	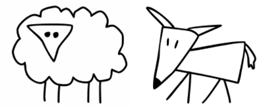	To expel the placenta	xxx
						Braided and tied on the neck	E	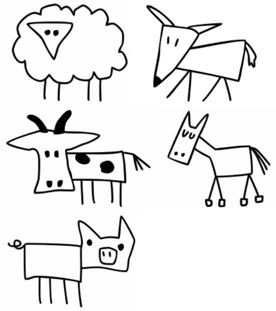	Anti-parasitic	xxx
			Spa		Bark	Tied on the leg	E	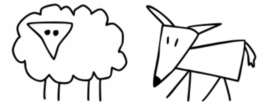	To treat broken paws	xx
			Spa			Tied in the animal's tail (ritual)	E	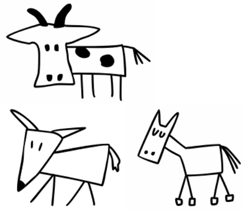	Anti-diarrheal	x
Deverra triradiata Hochst. ex Boiss M 20	Apiaceae		Egy	W	Aerial parts	Internal	Decoction	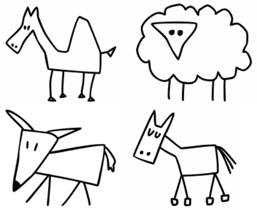	Digestive	x

Dittrichia graveolens (L.) Greuter ETH004	Asteraceae	Stinkwort	Gre	W	Aerial parts	Rubbed externally	E	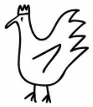	To treat lice and other bugs	xx
Dittrichia viscosa (L.) Greuter ETH004	Asteraceae	Sticky fleabane	Spa	W	Aerial parts	Decoction	E	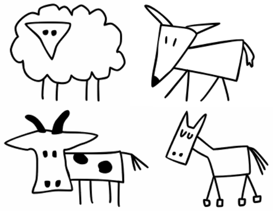	To treat wounds	xx
Dorycnium rectum (L.) Ser. GDA 49148	Fabaceae		Spa	W	Aerial parts	Decoction	E	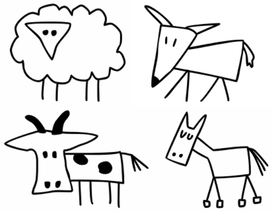	To treat wounds and burns	xx
Equisetum arvense L. BAGN52M	Equisetaceae	Horsetail	Ita	W	Aerial parts	Fodder	I	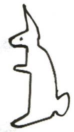	To strengthen their bones	x
Euphorbia characias L. and *E. dendroides* L ETH0045/ ETH045	Euphorbiaceae	Mediterranean and woody spurge	Gre	W	Latex	One to two drops of the latex in drinking water	I	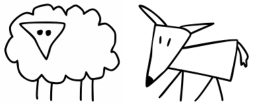	Digestive	x
Euphorbia helioscopia L. LEP-EUP	Euphorbiaceae	Madwoman's milk	Alb	W	Aerial parts	Fodder	I	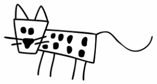	Self-medication for cats that have been bitten by snakes	x
Euphorbia hirsuta L. GDA 49227	Euphorbiaceae	Hairy spurge	Spa	W	Aerial parts	Decoction	E	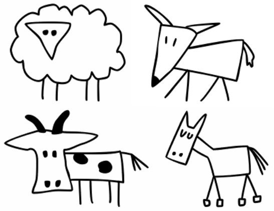	To treat wounds	x

Fagus sylvatica L. LEP-FAG	Fagaceae	Beech	Alb	W	Branches	Used for beating on the ears of the animal	E	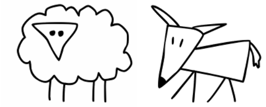	To treat unidentified diseases	xx
Foeniculum vulgare Mill.	Apiaceae	Fennel	Mor	W AND C	Whole plant	Decoction	I	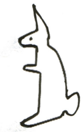	Digestive	xxx
			Mor		Fruit	Fodder	I	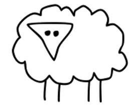	Galactagogue	xxx
			Alg		Fruit	Fodder	I	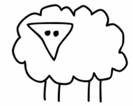	Galactagogue	xxx
Fraxinus excelsior L.	Oleaceae	Ash	Mor	W	Branches	Branches are suspended in the cattle shed	E	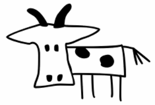	Protective against viper bites	x
Fraxinus ornus L. ETH071	Oleaceae	Manna ash	Gre	W	Leaves	Decoction	I	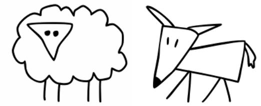	Digestive	x
Globularia arabica Jaub. & Spach M 06	Globulariaceae	Arabian Globularia	Egy	W	Whole plant	Topic application	E	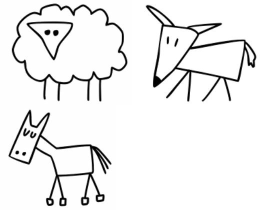	To heal wounds	x
			Egy		Whole plant	Decoction	I	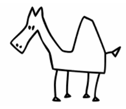	Digestive	x

Helichrysum stoechas (L.) Moench GDA 49144	Asteraceae	Everlasting	Spa	W	Aerial parts	Decoction	E	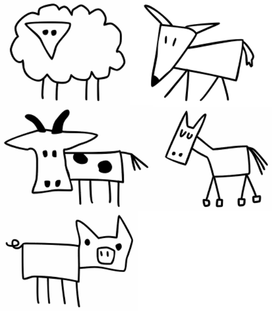	To heal wounds	x
Heracleum sphondylium L. LEP-HER	Apiaceae	Cow parsnip	Alb	W	Aerial parts	Fodder	I	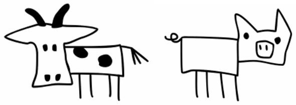	To strengthen pigs and as a galactagogue for cows	xx
Hypericum maculatum Crantz LEP-HYP	Guttiferae	Imperforate St. John's wort	Alb	W	Arial parts	Decoction	I	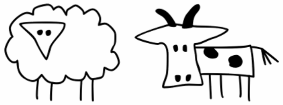	To treat digestive troubles and anti-diarrhea (sheep); antihelmintic (calves)	xx
Hypericum perforatum L. GDA 49068	Guttiferae	St: John's wort	Spa	W	Flowering tops	Macerate or fried in olive oil	E	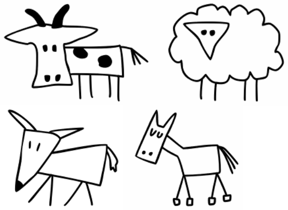	To treat wounds	xxx
			Spa			Maceration	E	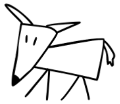	To heal mastitis	xxx
			Spa			Decoction	E	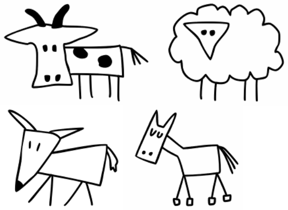	To treat bruises	x

			Mor			Macerate in oil	E	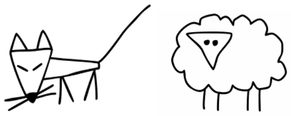	To treat skin disorders and bites	xx
Juglans regia L.	Juglandaceae	Walnut tree	Mor	SC	Leaves	Decoction, in external washes	E	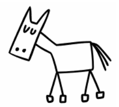	To prevent flies	xx
Juniperus communis L.	Cupressaceae	Juniper	Mor	W	Fruits	Macerated with anise fruit and soda	I	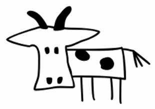	Digestive	xxx
Juniperus oxycedrus L.	Cupressaceae	Prickly juniper	Mor	W	Fruits	Macerated in alcohol together with soap powder	I	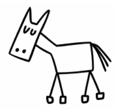	Digestive	x
Juniperus phoenicea L. CuJph01	Cupressaceae	Phoenician juniper	Alg	W	Leaves	Decoction	I	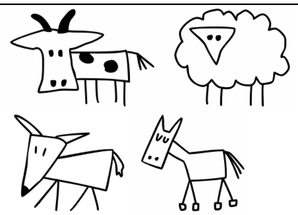	To treat respiratory diseases	xxx
Laurus nobilis L.	Lauraceae	Bay tree	Ita	SC	Leaves and fruits	Crushed and cooked in pork fat and applied topically. This product may be preserved, but it needs to be warmed up before it can be used	E	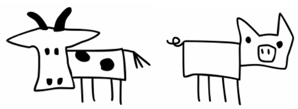	To treat contusions and skin inflammations	xx
Lavandula angustifolia Mill.	Lamiaceae	Lavender	Mor	C	Flowering tops	Rubbed on externally	E	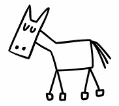	To treat local pains	x
Lawsonia inermis L. LyL.in01	Lythraceae	Henna	Alg	C	Leaves	Ground to a powder and added to fodder	I	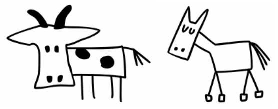	To treat constipations	xxx

			Mor		Leaves	Ground to a powder, mixed with water and rubbed on externally	E	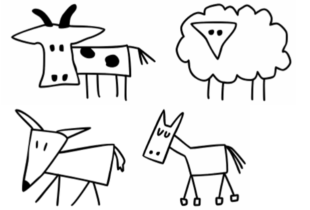	To treat skin diseases	xxx
Lilium martagon L. LEP-LIL	Liliaceae s.l.	Turk's cap lily	Alb	W	Bulbs	Decoction	I	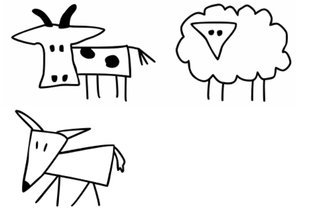	To treat liver diseases	xx
Linum usitatissimum L.	Linaceae	Flax	Ita	C	Seeds	Decoction	I	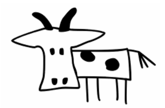	Emollient	xxx
Lycium shawii Roem.& Schult. M 24	Solanaceae	Desert thorn	Egy	W	Aerial parts	Topic application	E	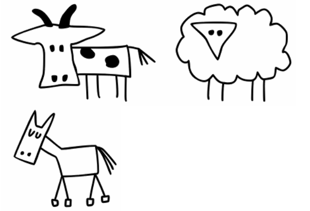	To treat skin inflammations	xxx
Malva sylvestris L. GDA 49154	Malvaceae	Mallow	Spa	W	Leaves	Decoction	E	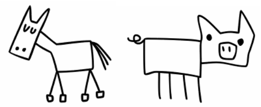	To treat pimples	x
			Alg		Leaves	Crushed and applied topically	E	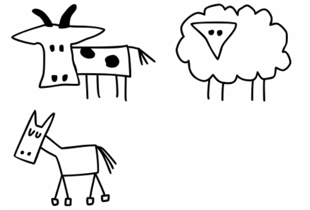	Haemostatic	xxx
Marrubium vulgare L. GDA 49076	Lamiaceae	White horehound	Spa	W	Aerial parts	Decoction	E	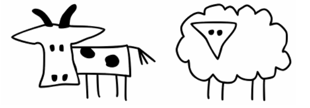	To treat wounds	x

								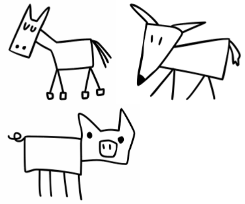		
Matricaria chamomilla L ARI 5865	Asteraceae	Chamomile	Cyp	W	Flowering tops	Ground to a powder , and applied topically	E	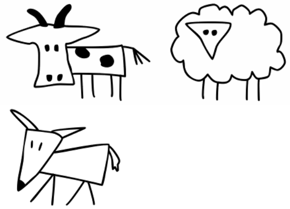	To treat skin inflammations (especially to kill fleas)	xxx
Mentha longifolia (L.) Huds. ETH072	Lamiaceae	Horsemint	Gre	W	Leaves	Decoction	I	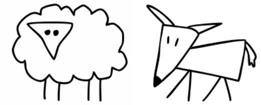	Digestive	xxx
Mentha x *piperita* L.	Lamiaceae	Peppermint	Mor	C	Leaves	Decoction	I	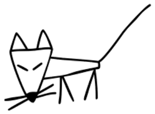	Digestive	xxx
Mentha spicata L. ETH073	Lamiaceae	Spearmint	Gre	W	Leaves	Decoction	I	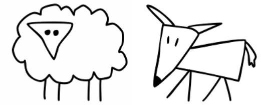	Digestive	xxx
Mercurialis annua L.	Euphorbiaceae	Mercury	Mor	W	Roots	Decoction	I	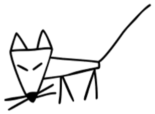	To treat respiratory diseases	xx
Nerium oleander L. ApNol01	Apocynaceae	Oleander	Alg	W	Leaves	Decoction, used in fumigation	E	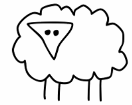	Thought to evacuate parasites from the brain	xxx
Oenanthe pimpinelloides L. CAP55M	Apiaceae	Meadow parsley	Ita	W	Leaves	Fodder	I	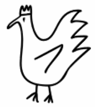	To treat stomach's swelling in chicken	xxx

Olea europaea L.	Oleaceae	Olive tree	Cyp	C	Fruits→Oil	Mixed with *Agaricus* sp., and rubbed on externally	E	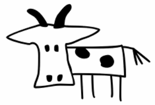	To treat skin disorders: and neck wounds	xxx
			Cyp			As beverage	I	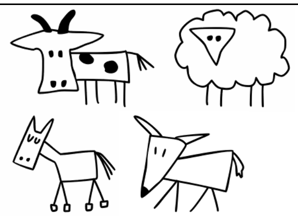	Digestive, as an antidote for food poisoning	xxx
			Cyp			Mixed with wine, and rubbed on externally	E	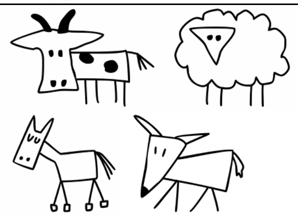	Antiseptic	xxx
Olea europaea L. and *O. europea* L. ssp. o*leaster* ETH076		Olive tree and wild olive tree	Gre	C AND W	Leaves	Decoction	E	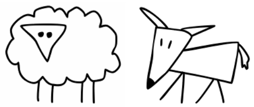	To heal eye infections	xx
Onopordum sp. GDA 49263	Asteraceae	Cotton thistle	Spa	W	Root	Decoction	E	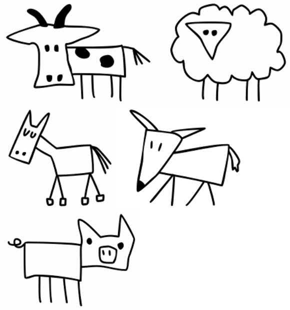	To treat wounds	x
Origanum floribundum Munby LaOfl01	Lamiaceae		Alg	W	Leaves	Added to the feed	I	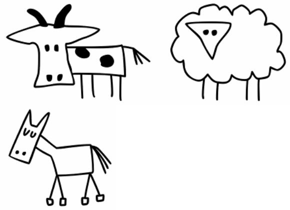	Appetizer	xxx

Origanum vulgare L. ssp. *hirtum* Ietswaart ETH063	Lamiaceae	Greek oregano	Gre	W	Leaves	Decoction	I	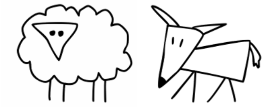	Digestive	xxx
Pallenis spinosa (L.) Cass. GDA 49062	Asteraceae		Spa	W	Aerial parts	Decoction	E	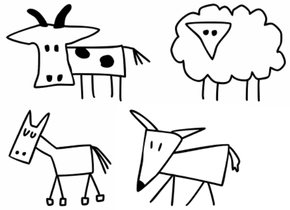	To heal wounds	x
Parietaria diffusa M. et K. CAP56M	Urticaceae	Pellitory of the wall	Ita	W	Aerial part	Fodder	I	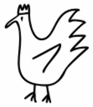	To cures the stomach's swelling	xxx
Peganum harmala L. M 27	Zygophyllaceae	Syrian rue	Egy	W	Whole plant	Decoction	I	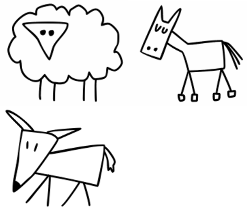	Digestive	xxx
Petroselinum crispum (Miller) A.W. Hill.	Apiaceae	Parsley	Mor	C	Aerial parts	Applied topically	E	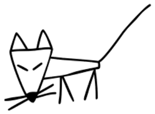	To relief breast inflammations during weaning	xxx
Phlomis purpurea L. GDA 49108	Lamiaceae	Jerusalem sage	Spa	W	Aerial parts	Applied topically	E	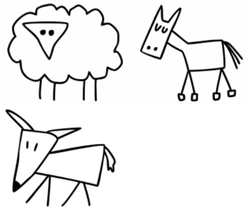	To treat diarrhoea	x
Pinus sylvestris L.	Pinaceae	Scots pine	Mor	W	Tar	Applied topically on the muzzle	E	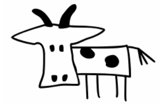	Preventive	xx

Pistacia lentiscus L.<br>ARI 5625,<br>ARI 5693	Anacardiaceae	Mastix tree	Cyp	W	Leaves and stems	Chewed by a person and then expelled into ox's eyes	E	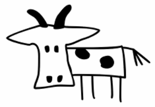	To treat eye inflammations	xxx
			Alg	W	Leaves	Decoction	I	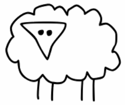	Digestive	xxx
Plantago lanceolata L.	Plantaginaceae	Narrow-leaved plantain	Mor	W	Leaves	Topic application	E	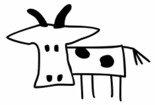	To treat insect bites	xx
Plantago major L.<br>CAP58M	Plantaginaceae	Common plantain	Ita	W	Leaves	Topic application	E	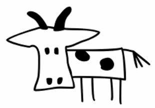 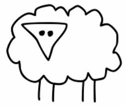	To cure skin's infections	xx
Polygonum aviculare L.<br>BAGN34M	Polygonaceae	Knotweed	Ita	W	Aerial parts	Fodder	I	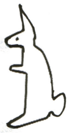	To treat diarrhoea	x
Potentilla reptans L.<br>BAGN54M	Rosaceae	Cinquefoil	Ita	W	Aerial parts	Fodder	I	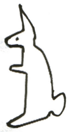	To treat diarrhoea	x
Prunus dulcis (Miller) D. A. Webb. var. amara	Rosaceae	Bitter almond	Mor	C	Kernels	Beverage	I	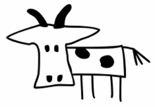	Purgative	xxx
Prunus domestica L.<br>(Rosaceae)<br>LEP-PRU	Rosaceae	Wild plum	Alb	SC	Fruits→distillate	Topic application	E	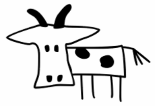 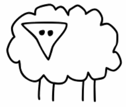 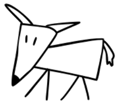	To treat wounds in animals	xxx

Pteridium aquilinum (L.) Kuhn.	Pteridiaceae	Bracken fern	Alb	W	Aerial parts	Put in stables	E	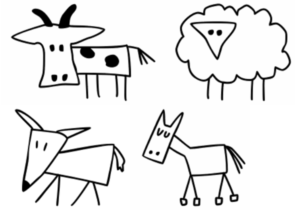	Antiseptic	xx
Pulicaria odora (L.) Reichenb GDA 49066	Asteraceae		Spa	W	Flowering tops	Macerated in alcohol	E	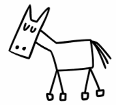	To heal wounds and bruises	xx
			Spa			Decoction	E	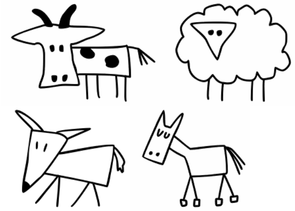	To treat wounds	xx
Quercus lusitanica Lam.	Fagaceae	Lusitanian oak	Mor	W	Gall or seeds	Fodder	I	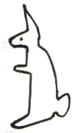	Digestive	x
Quercus rubra L.	Fagaceae	Red oak	Mor	W	Branches	Fodder	I	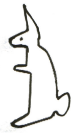	Anti-diarrhoea	xv
Retama raetam (Forssk.) Webb. & Berthel M 29	Fabaceae	White weeping broom	Egy	W	Aerial parts	Internal	I	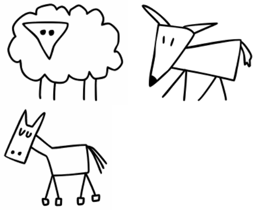	Digestive	xxx
Rhamnus alaternus L. RhRa01	Rhamnaceae	Evergreen buckthorn	Alg	W	Leaves	Decoction	I	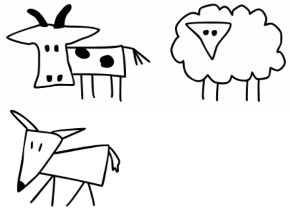	To treat stomach swelling	xx

Rhamnus alpinus L.<br>LEP-RHA	Rhamnaceae	Alpine buckthorn	Alb	W	Aerial parts	Decoction, used in washes	E	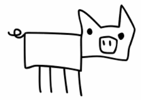	To heal eczemas	x
Rorippa nasturtium-aquaticum (L.) Hayek<br>CAP53M	Brassicaceae	Watercress	Ita	W	Aerial parts	Decoction	I	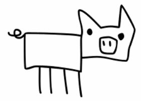	Diuretic	xxx
Rosa arabica Crép.<br>M 30	Rosaceae	Sweet briar rose	Egy	W	Whole plant	Decoction	I	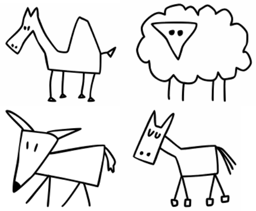	To treat reproductive troubles	x
Rosmarinus officinalis L.	Lamiaceae	Rosemary	Mor	C	Flowering tops	Decoction, in external baths	E	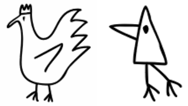	To treat "fever" (parasites?)	xx
Rumex acetosa L.	Polygonaceae	Sorrel	Ita	W	Aerial parts	Fodder	I	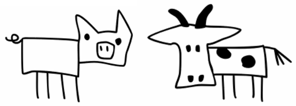	Laxative	x
Ruta chalepensis L.<br>ARI 5565, ARI 5604	Rutaceae	Egyptian rue	Cyp	W	Aerial parts	Ground to a powder and dissolved in water	I	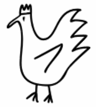	To prevent microbic infections	xxx
					Aerial parts	Rubber externally on wounds		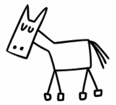	To bind flies and prevent infections	x
ETH074			Gre	W	Leaves	Decoction	I	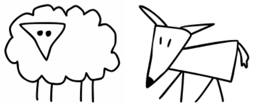	Digestive	xx

Salvia officinalis L.	Lamiaceae	Sage	Mor	C	Aerial parts	Decoction	I	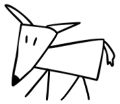	To treat respiratory troubles	xx
Sambucus nigra L. ARI 5619	Caprifoliaceae	Elderberry tree	Cyp	C	Inflorescence	Decoction	E	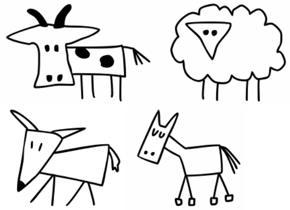	To treat eye inflammations and ski disorders (also as a preventive)	xx
Sanguisorba minor L. LEP-SAN	Rosaceae	Salad burnet	Alb	W	Aerial parts	Cut, mixed with salt, and then rubbed on the udder	E	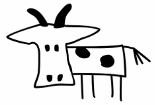	To heal mastitis	x
Senecio jacobaea L. GDA 49130	Asteraceae		Spa	W	Aerial parts (including flowers)	Decoction	E	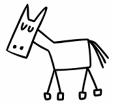	To treat wounds	xxx
			Spa			Macerate in olive oil and alcohol	E	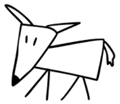	To treat mastitis	x
Tanacetum santolinoides (DC.) Feinbrun & Fertig M 40	Asteraceae		Egy	W	Aerial parts	Decoction	I	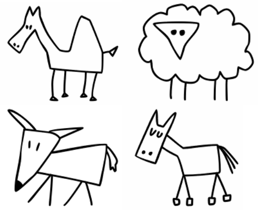	Digestive	x
Taraxacum laevigatum DC. AsTla01	Asteraceae	Red-seed dandelion	Alg	W	Leaves	Fodder	I	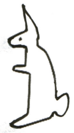	Laxative	xxx
Thymus vulgaris L.	Lamiaceae	Thyme	Mor	W	Flowering tops	Decoction	E	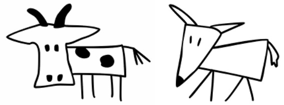	To heal wounds, mouth and udder inflammations	xxx

								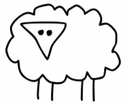		
Trigonella foenum- graecum L.	Fabaceae	Fenugreek	Mor	C	Seeds	Fodder	I	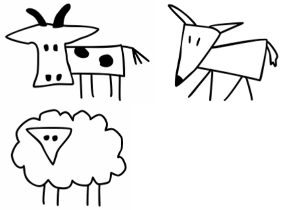	To enhance the quality of the hair	xxx
			Alg	C	Seeds	Fodder	I	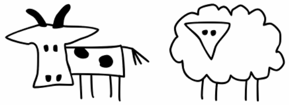	To strengthen the animal (increasing its weight); especially administrated before the animal is sold	xxx
Tuberaria lignosa (Sweet) Stamp. GDA 49158	Cistaceae		Spa	W	Aerial part	Decoction	E	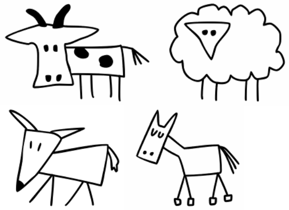	To treat wounds	xxx
Verbena officinalis L. GDA 49140	Verbenaceae	Vervain	Spa	W	Leaves	Decoction	E	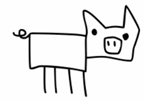	Antiseptic after castration	x
Zea mays L.	Poaceae	Corn	Ita	C	Seeds	The seeds are decocted in water and/ or milk; the decoction is applied externally	E	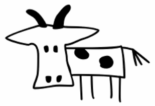	To treat mastitis	x

Alb: Albania; Alg: Algeria; Cyp: Cyprus; Egy: Egypt; Gre: Greece; Ita: Italy; Mor: Morocco; Spa: Spain; C: cultivated; SC: semi-cultivated; W: wild; E: external; I: internal;
x: rare use (use quoted by less than 10% of the informants); xx: common use (use quoted by more than 10% and less than 40% of the informants); xxx: very common use (use quoted by more than 40% of the informants)

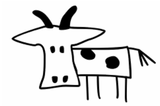 cattle 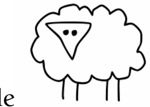 sheep 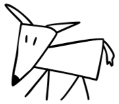 goats 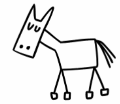 equines 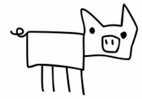 swine 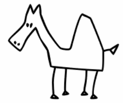 camels 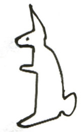 rabbits 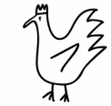 poultry 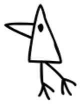 birds

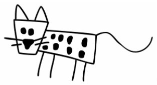 cats 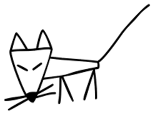 dogs 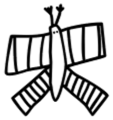 bees
